# Development and Relative Validity of a Food Frequency Questionnaire to Assess Intakes of Total and Free Sugars in Australian Toddlers

**DOI:** 10.3390/ijerph14111361

**Published:** 2017-11-08

**Authors:** Gemma Devenish, Aqif Mukhtar, Andrea Begley, Loc Do, Jane Scott

**Affiliations:** 1 School of Public Health, Curtin University, Perth 6102, Australia; gemma.devenish@curtin.edu.au (G.D.); Aqif.Mukhtar@curtin.edu.au (A.M.); a.begley@curtin.edu.au (A.B.); 2 Australian Research Centre for Population Oral Health, The University of Adelaide, Adelaide 5000, Australia; loc.do@adelaide.edu.au

**Keywords:** food frequency questionnaire, dietary assessment, validation, early childhood caries, pre-school, sugar, free sugars

## Abstract

*Background*: Dental research into early childhood caries is hindered by a lack of suitable dietary assessment tools that have been developed and validated for the population and outcomes of interest. The aim of this study was to develop and investigate the relative validity and reproducibility of the Study of Mothers’ and Infants’ Life Events Food Frequency Questionnaire (SMILE-FFQ), to assess the total and free sugars intakes of Australian toddlers. *Methods*: The SMILE-FFQ was designed to capture the leading dietary contributors to dental caries risk in toddlers aged 18–30 months via a proxy report. Ninety-five parents of Australian toddlers completed the questionnaire online before and after providing three 24-h recalls (24HR), collected on non-consecutive days using the multipass method. Total and free sugars were compared between the two SMILE-FFQ administrations and between each SMILE-FFQ and the 24HR using multiple statistical tests and standardised validity criteria. Correlation (Pearson), mean difference (Wilcoxon rank test) and Bland Altman analyses were conducted to compare absolute values, with cross-classification (Chi-Square and Weighted Kappa) used to compare agreement across tertiles. *Results*: All reproducibility tests showed good agreement except weighted kappa, which showed acceptable agreement. Relative validity tests revealed a mix of good and acceptable agreement, with total sugars performing better at the individual level than free sugars. Compared to the 24HR, the SMILE-FFQ tended to underestimate absolute values at lower levels and overestimate them at higher levels. *Conclusions*: The combined findings of the various tests indicate that the SMILE-FFQ performs comparably to the 24HR for assessing both total and free sugars among individuals, is most effective for ranking participants rather than determining absolute intakes, and is therefore suitable for use in observational studies of Australian toddlers.

## 1. Introduction

Dental diseases are the most prevalent non-communicable disease globally [1] and the leading cause of preventable hospitalisations among children in Australia [2]. The prevalence of dental caries in Australian children began to decline significantly during the 1970s. However, in the mid-1990s, this downward trend reversed and caries prevalence has increased steadily ever since [3,4]. In addition, disparities in caries experience exist between children based on socio-economic status, remoteness and availability of fluoridated water [4].

The relationship between free sugar intakes and dental caries is well established, although the lower level of the effect is less clear [1,5,6,7]. In 2015, the World Health Organization (WHO) released the *Guideline: Sugars intake for adults and children*, with a focus on preventing dental disease and obesity [1]. Although the Guideline sets out a strong recommendation to reduce the intake of free sugars to less than 10% of total energy intake, the conditional recommendation to further reduce intake to less than 5% of total energy was limited by the low quality of evidence. The supporting evidence review highlights the lack of consistency and precision of dietary assessment methods in dental studies [5], leading to a call within the Guideline for new studies with an improved dietary assessment methodology [1].

Controlling for key dietary risk factors is a critical component of oral health research. However, dietary assessment is complex, and reviews of dental studies highlight the inconsistency and imprecision of approaches used for measuring dietary intakes and food behaviours [6,8,9]. Newly designed dietary assessment tools should be validated prior to use, and existing tools should be calibrated to each cohort [10,11,12]. An ongoing limitation of dental research is the lack of rigour in dietary assessment methodology, with many studies still not using validated dietary questionnaires to capture sugars intake, and fewer still undertaking internal calibration [5,8]. 

One reason for this lack of rigour is that an appropriate tool often does not exist [8,13]. Dietary assessment design and validation is a branch of nutrition research, and dental research teams do not always have the resources or nutrition expertise required to undertake validity studies. Childhood dietary intake is particularly challenging to capture, due to the participant age and varying ability to self-report intake, as well as the greater rate of fluctuation in dietary intake patterns throughout childhood compared to adults [10,14]. This is especially relevant throughout the first three years of life, as a child progresses from a newborn diet of breast milk or infant formula, via a range of textures, through to family foods. This transitioning diet combined with the unique dietary data requisites of dental research means that the tools used during the pre-school years need to be age-specific and developed purposely for dental research.

There are a number of methods used in dietary assessment, but the Food Frequency Questionnaire (FFQ) is considered the most appropriate data collection method for large, prospective studies [10,11,12]. Once developed, it is quick and inexpensive to administer and process, as it can be self-administered and rapidly analysed. Additionally, it has a lower subject burden than weighed food records, and captures usual dietary intakes over a longer period of time [10,11]. Although it is a commonly used method, the FFQ must be tailored to both the population and outcomes of interest if it is to produce useful data [10,11,12].

The aim of this study is twofold: firstly, to develop a FFQ to assess intakes of total and free sugars from major food and beverage sources, fluoride from non-water sources and other key foods relevant to dental caries in Australian toddlers aged 18–30 months via a proxy report. Secondly, to investigate the relative validity and reproducibility of this FFQ for total and free sugars against repeat 24-h recalls (24HR).

## 2. Materials and Methods

### 2.1. Study of Mothers’ and Infants’ Life Events Affecting Oral Health (SMILE)

The Study of Mothers’ and Infants’ Life Events Affecting Oral Health (SMILE) is a longitudinal birth cohort study that aims to identify and evaluate the relative importance and timing of critical factors that shape the oral health of young children and then evaluate those factors in terms of their interrelationship with socioeconomic influences [15]. A limited number of dietary assessment tools have been used to assess young children’s total dietary intake [16,17,18,19]; however, a tool for the dietary assessment of dental significance through the toddler years was not available. As such, a FFQ was designed to capture the leading dietary contributors to dental caries risk in toddlers aged 18–30 months via a proxy report for use in SMILE.

#### SMILE Food Frequency Questionnaire Development

Development of the SMILE-FFQ occurred in four stages, following the methods described by Willett [11] and Coulston et al. [20]. Firstly, a brief review of the literature identified the leading dietary contributors to dental caries risk, namely major food and beverage sources of total sugars, free sugars and fluoride, as well as protective foods including cheese and other milk products, chewing gum and xylitol [6]. The World Health Organization definition of free sugars was used, which “include(s) monosaccharides and disaccharides added to foods and beverages by the manufacturer, cook or consumer, and sugars naturally present in honey, syrups, fruit juices and fruit juice concentrates” [1].

Secondly, a food list was generated, incorporating all foods that would provide these dietary contributors. This list was then refined to exclude food items that: (a) contained only a small amount of the nutrient in question (for example, commercial pasta sauce containing approx. 5% total sugar); or (b) are not typically consumed by individuals within this age group (for example, chewing gum, alcohol, coffee, and energy drinks). During this process, foods were collapsed into line-items based on similarity of the food and of total and free sugar content and then grouped into blocks by food type. The final list (Supplementary Materials) consisted of 24 question blocks containing 89 line-items. Four additional questions were added to further split the coding of some food items (for example, “If your child eats yoghurt, do you choose reduced fat versions?”) and two questions were included to capture eating behaviours of dental significance (usual drinking vessels and typical number of eating occasions per day).

Thirdly, frequency and quantity response options appropriate to this age group were developed for each line-item. Seven frequency response options were used for all line-items, commencing with “never or rarely” before ranging from “1 time every 2 weeks” to “3 or more times per day”. Quantity response options were tailored to each question block, using a combination of household measures (teaspoon, tablespoon, cup) and typical portion sizes (piece, tub, pouch, etc.). Weight (g) or volume (ml) was also provided, except in blocks which only used teaspoon and tablespoon options, such as condiments.

Finally, a database was developed to analyse the SMILE-FFQ, linking scoring algorithms for all possible responses to nutrient values for total and free sugars, derived from representative foods in the AUSNUT 2011–13 food composition database [21].

The SMILE-FFQ was produced online using Qualtrics software, 2014 version (Qualtrics, Utah, USA, www.qualtrics.com). A copy of the questionnaire is provided in Supplementary Materials.

### 2.2. Food Frequency Questionnaire Validation

Curtin University Human Research Ethics Committee approved the study (Approval No. SPH-39-2014). The validation study was registered with the US National Cancer Institute Dietary Assessment Calibration/Validation (DACV) Register (Study ID 0293).

#### 2.2.1. Participants and Recruitment

Inclusion criteria were parents with a child aged between 18 and 30 months at the time of recruitment, who lived in Australia and who had access to the internet, an email address, and telephone. No other inclusion or exclusion criteria were identified at this stage; however, one parent later withdrew from the study as they were unable to report their child’s dietary intake on any weekday.

**Figure 1:**
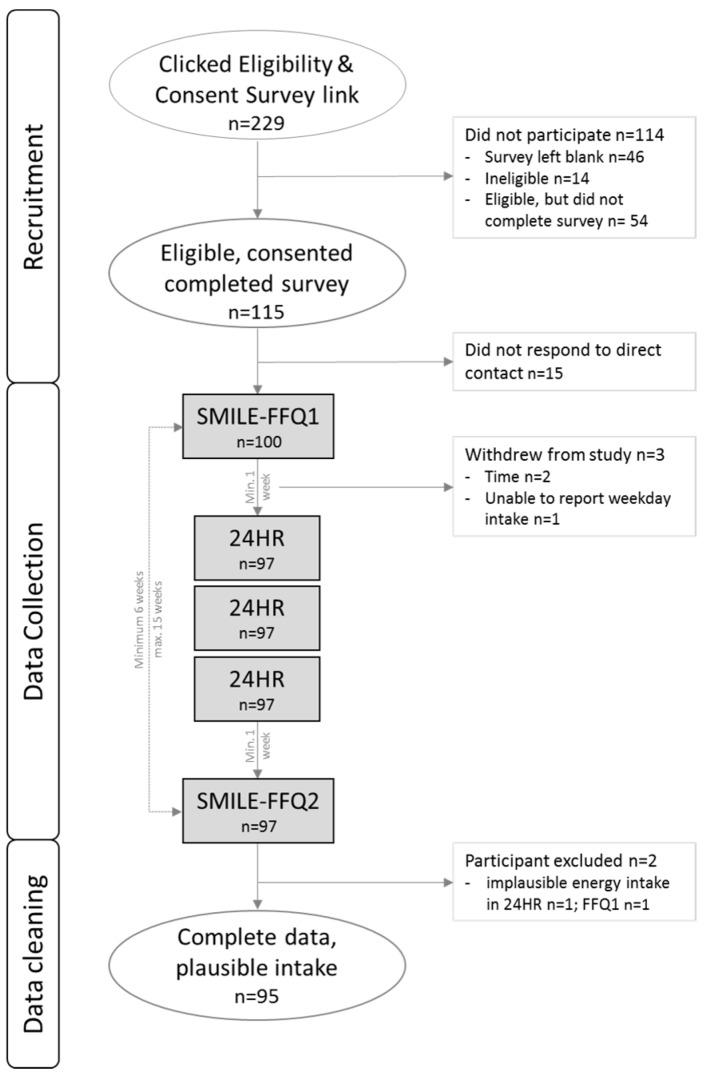
Recruitment and data collection for the SMILE-FFQ validation study. SMILE-FFQ: Study of Mothers’ and Infants’ Life Events Food Frequency Questionnaire; 24HR: 24-h recalls.

A Facebook page was created to recruit participants, with a target sample size of 100, in line with recommendations by Willett [11] and comparable to similar studies [22,23,24,25]. The page included study information and a post with a link to an expression of interest, eligibility, and consent survey. The first page of the survey asked participants to report against the inclusion criteria. Those not meeting the eligibility requirements were diverted to an exit page, while eligible participants continued to the consent section. The survey was closed two weeks after the page launched, with a reach of 1753 people who saw the post, 229 who clicked the link, and 115 eligible participants who completed the survey (Figure 1). Fifteen people who completed the eligibility survey did not respond to any follow up communication or questionnaires, so the final number recruited was 100. Three participants withdrew after the first questionnaire, and the remaining 97 completed the study.

#### 2.2.2. Data Collection

After recruitment, participants were entered into the study in three waves between August and October, to allow a single researcher to conduct all telephone diet recalls. Participants that were in the second or third wave were informed of the delay and the expected date of commencement. Data collection for all participants occurred between August and December, with one participant’s final FFQ completed in early January (the greatest duration from commencement at a total of 15 weeks).

Data collection, depicted in Figure 1, occurred over a minimum six-week period for each participant. Parents were sent a link to the online questionnaire (SMILE-FFQ1) and followed-up via weekly reminder emails which contained an unsubscribe option. One week after SMILE-FFQ1 was completed, participants entered the 24-h diet recall phase (24HR), consisting of three recalls conducted via telephone on non-consecutive days by the principal researcher (GD) who is a dietitian. A random number generator was used to allocate three days within a four week period for the participant recalls; two weekdays and one weekend day. On the day of a recall, participants were sent an SMS to inform them they would be receiving a call and were able to nominate a suitable time of day for the call. If the child had been unwell or the parent was unaware of intake on the previous day (e.g., child in childcare), the recall was not conducted, and a new day was randomly allocated. If the parent was able to report intake despite the child being in the care of others, the allocated recall day was retained. The five-step multipass method [26] was used to conduct the 24HR, using a modified forgotten foods list (the question about alcohol was substituted for “any rusks or teething foods?”) and a uniquely designed probing protocol with specific questions for toddler foods in the detail cycle phase. One week after the final 24HR, and at least six weeks after SMILE-FFQ1, participants were sent a new link to the online questionnaire (SMILE-FFQ2). Demographic data were collected on the final page of the SMILE-FFQ2, with the child’s date of birth also captured in the eligibility survey.

The 24HR were entered into FoodWorks version 8 (Xyris Software, 2012–2017, Brisbane, Australia) using the current Australian food composition database, AUSNUT 2011–13. As FoodWorks does not provide free sugars values, these were later imputed in Microsoft Access version 15 (Microsoft Corporation, 2013, Washington, DC, USA) using a dataset developed to accompany AUSNUT 2011–13. This dataset was developed by Food Standards Australia and New Zealand using the methodology by Louie et al. [27], which employed the WHO definition of free sugars [1]. Mean daily intakes for total and free sugars were obtained for each participant. Descriptive statistics were used to identify outliers for the weight of food and energy consumed and the data were checked for entry errors and resolved. 

The survey responses for the SMILE-FFQs were exported from Qualtrics in Excel format and linked to the scoring database using Microsoft Access. Mean SMILE-FFQ1 and SMILE-FFQ2 daily intakes of total and free sugars were then generated for each participant.

Two participants were identified as reporting implausibly high intakes of both energy and total sugars and were excluded from the analysis. Implausible intake was defined as values greater than 3 SD above the mean for both nutrients in at least one questionnaire [28]. 

#### 2.2.3. Data Analysis

Data were exported from Microsoft Access to Microsoft Excel and then analysed using SPSS version 24 (IBM SPSS Statistics for Windows, New York, NY, USA). The data were not normally distributed, so natural log transformation was performed and used with all but the Bland Altman analyses. The data were not energy adjusted, as the SMILE-FFQ was not designed to assess total diet and therefore total energy intakes could not be obtained.

As recommended by Lombard et al. [29], multiple statistical tests were employed to assess different facets of validity at both the group and individual level. Correlation (Pearson) and Cross-classification (Chi-Square and Weighted Kappa) assessed validity at the individual level. Comparison of means (Wilcoxon rank test) compared agreement between the two tools at a group level. Bland-Altman analyses of the raw data, including calculation of the Limits of Agreement and Bland Altman Index, investigated individual differences from the group mean and the extent of bias at a group level. Linear regression analysis was undertaken to investigate whether the slope of mean bias was significantly different from zero, indicating proportional bias.

Outcome cut-offs were determined using criteria described in Lombard et al. [29]. For correlation coefficient (Pearson), good agreement was ≥0.50, acceptable from 0.20 to 0.49, and poor <0.20 [29,30]. For cross-classification (Chi Square), good agreement was when ≥50% of participants were similarly classified (same tertile) and ≤10% dissimilarly classified (opposite tertile); and poor agreement was when <50% were similarly classified and/or >10% dissimilarly classified [29,30]. For the Weighted Kappa, good agreement was ≥0.61, acceptable from 0.20 to 0.60, and poor <0.20 [29,30]. For comparison of means (Wilcoxon), good agreement was at *p* > 0.05 and poor was *p* ≤ 0.05. A Bland Altman Index score of ≤5% was considered good agreement [31,32].

## 3. Results

### 3.1. Participant Characteristics

Data from 95 participants were analysed. Respondents were predominantly university-educated mothers, with a mean age of 34.2 ± 4.4 years at the time of the study (Table 1).

### 3.2. Total and Free Sugars Intakes

Table 2 provides mean values of total and free sugars in grams per day as measured by the SMILE-FFQ1, SMILE-FFQ2, and 24HR, along with further descriptive statistics and measures of normality before and after log transformation. SMILE-FFQ1 recorded the highest mean intake and the widest range of responses for both total and free sugars.

### 3.3. Relative Validity of the SMILE-FFQ

Relative validity was assessed by comparing total and free sugars values obtained by 24HR to those determined by SMILE-FFQ1 and SMILE-FFQ2. Agreement at the individual level (Table 3) was either good (Chi-Square, Pearson for total sugars) or acceptable (Weighted Kappa, Pearson for free sugars). Cross-classifications demonstrated good agreement between the two methods in classifying participants into tertiles, with less than 10% of participants misclassified into an opposite tertile for all methods except free sugars SMILE-FFQ2, which was just over at 10.6%.

Comparison at the group level (nonparametric) found no significant difference in mean intake between each pair of methods, except for the difference in the mean intake of total sugars between SMILE-FFQ1 and 24HR (Table 4).

The Bland Altman Index showed either 5.3% or 7.4% of participants falling outside of the limits of agreement, with total sugars close to the acceptable cut-off of 5%. The limits of agreement appear to be wide when compared to the mean, SD, and range of reported intakes (Table 2), and were greatest for total sugars between SMILE-FFQ1 and 24HR.

**Figure 2:**
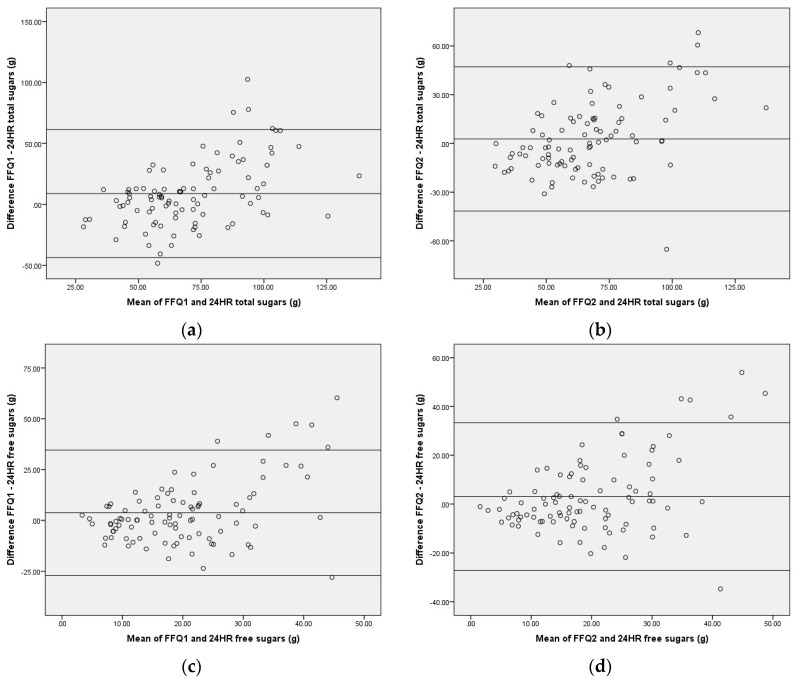
Bland Altman Plots comparing total and free sugars measured by the SMILE-FFQ compared with repeat 24-h recalls: (**a**) Total Sugars FFQ1 compared with 24HR; (**b**) Total Sugars FFQ2 compared with 24HR; (**c**) Free Sugars FFQ1 compared with 24HR; (**d**) Free Sugars FFQ2 compared with 24HR. FFQ 1 and 2 refers to the same tool (SMILE-FFQ) administered before (FFQ1) and after (FFQ2) the recalls.

Visual inspection of Bland-Altman plots suggested a positive slope and a tendency to increase in variability as the magnitude of the measure increases (Figure 2). Linear regression verified that the slope of bias was significantly different from zero for all four measures (*p* < 0.001), indicating that proportional bias was present. These results suggest that agreement between the SMILE-FFQ and 24HR was better amongst participants with moderate total and free sugars intakes, but the SMILE-FFQ tended to underestimate sugars for participants with lower reported 24HR intakes and overestimate sugars for participants with higher 24HR intakes.

### 3.4. Reproducibility of the SMILE-FFQ

Reproducibility was assessed by comparing total and free sugars values obtained by SMILE-FFQ1 and SMILE-FFQ2. Agreement at the individual level (Table 5) was either good (Chi-Square, Pearson) or acceptable (Weighted Kappa). Cross-classifications demonstrated good agreement between the two methods in classifying participants into tertiles, with less than 10% of participants misclassified into an opposite tertile for both total and free sugars.

Comparison at the group level found no significant difference between the two FFQs for mean free sugars intakes, indicating good agreement; however, for total sugars, there was a significant difference, indicating poor agreement (Table 6).

The Bland Altman Index showed either 5.3% or 6.3% of participants falling outside of the limits of agreement, with total sugars close to the acceptable cut-off of 5%. The limits of agreement appear to be wide when compared to the mean, SD, and range of reported intakes (Table 2).

**Figure 3:**
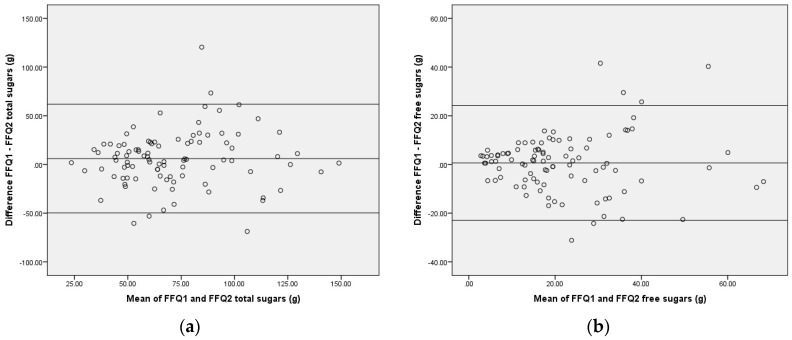
Bland Altman Plots comparing total and free sugars measured by repeat administrations of the SMILE-FFQ: (**a**) Total Sugars FFQ1 compared with FFQ2; (**b**) Free Sugars FFQ1 compared with FFQ2.

Bland-Altman plots demonstrated good agreement between repeat administrations of the SMILE-FFQ, as the data were scattered with no proportional bias (Figure 3). Linear regression also indicated that the slope of bias was not significantly different from zero for both measures (*p* > 0.01), suggesting there was no trend in differences in performance between the FFQs at varying levels of intake.

## 4. Discussion

### 4.1. Validity

Food Frequency Questionnaires aim to provide estimates of long-term dietary intake, and are generally considered unsuited to estimating absolute daily intakes of nutrients or capturing short-term dietary intakes [10,11]. The findings of this validation study reflect this, as the weakest agreement was observed in comparisons of absolute intake.

Combining the findings of the various tests, it is evident that individual level validity is more consistently rated as good than group level validity, and that the tool is most effective for ranking participants but not necessarily for determining absolute intakes of total and free sugars. The tendency of the tool to underestimate intakes in participants with lower 24HR values and overestimate intakes in those with higher values is consistent with other FFQs [23,33], and has a lesser impact on the accuracy of ranking than of absolute intakes. These results indicate that the SMILE-FFQ performs comparably to the 24HR as a measure of total and free sugars of individuals and to a lesser extent the group, and is therefore acceptable for use in observational studies of Australian toddlers if absolute sugars intakes is not an explanatory or outcome measure.

At present, the number of available tools which assess dietary intakes during early childhood is very small, and fewer still capture total sugars. To our knowledge, this is the first FFQ designed to measure free sugars in this age group (18–30 months), and the first Australian FFQ that has been validated for total or free sugars in any pre-school aged children (0–5 years). As such, comparison data in this age group are scarce. The National Infant Feeding Survey focuses on milk-feeding methods in the first two years of life rather than total diet [18], and although the recent Australian Health Survey included participants as young as two, reporting of results for this age group is incomplete [34].

Bell et al. [35] recently developed a short food-group-based dietary risk assessment tool for use with Australian toddlers (aged 1–3 years). This 19-item screener generates a dietary risk score between 0 and 100, but is not designed to obtain nutrient values. Flood et al. [25] developed a 17-item screener in FFQ style to rank Australian pre-school aged children (2–5 years) based on key dietary habits. Four questions pertained to major contributors of free sugars (biscuits, confectionery, soft-drink, fruit juice); however, two of these performed poorly against the reference method. Burrows et al. [36] developed the 70-item Australian Recommended Food Score for Pre-schoolers (ARFS-P), but to date, this has only provided validation reporting against the FFQ from which it was developed, and scoring occurs at the food group rather than nutrient level, with a focus on core foods.

A FFQ has been developed for 12-month-olds in the United Kingdom [22] and modified for use in New Zealand with toddlers aged 12–24 months [24,37]; however, it was not designed to capture free sugars, and the New Zealand validation studies do not report findings for total sugars. Nevertheless, it is rarely appropriate to use a tool designed for another population without adaptation and further validation, as food supply, composition, and terminology differ between countries. A notable example is the eight-item screener developed to assess the consumption of sugar snacks in Ugandan school-aged children (mean 12.4 years of age) [38]. This tool lists tea and coffee as two of the eight food items included and as such is not transferable to Australian children. Although tea and coffee are significant contributors to sugar consumption of Ugandan children, the 2007 Australian National Children’s Nutrition and Physical Activity Survey reports a very low prevalence of consumption of tea and coffee in this age group, at 6.6% and 2.3%, respectively [39], and so the tool is not compatible with Australian research.

### 4.2. Challenges in Dietary Assessment of Pre-School Aged Children

Dietary assessment in early childhood presents some unique challenges. Young children are unable to self-report, so proxy reporting must be used to collect the data from a surrogate [10,14,40,41]. Parents are not always aware of their child’s complete dietary intake as feeding roles may be shared amongst other caregivers and child-care providers [14,41]. In addition, toddlers tend to have higher amounts of plate waste than older children and adults, which makes estimating actual consumption more cognitively complex [10,14].

In addition, toddlers experience a unique food landscape. Portion sizes are small and highly variable, and the frequency of meals and snacks tends to be greater than for older children and adults. Young children also have strong appetite cues [42], and experience irregular patterns of rapid physical and cognitive growth. As a result, food intake and preferences have greater variability than that of adults in terms of both day-to-day intake and overall eating pattern, and change rapidly across the pre-school years [10,14,41]. An FFQ for this age needs to offer a wide range of lower-size portion options that include very small values. These small amounts seem negligible to overall nutritional intake; however, if lower end options are not provided, reporters may select the lowest available value, which may be several times larger than what was actually consumed. Also, due to the smaller energy and nutrient requirements of pre-school aged children, the small, frequent “tastes” of food may add up to a significant proportion of overall intake. The development of FFQs for this age bracket has often occurred by testing the validity of a tool originally designed for adults or older children, the portion sizes of which may be inappropriate and contribute to the common finding of overestimation [23,33,43,44].

These challenges limit the accuracy of most dietary assessment methods, including the SMILE-FFQ. The design of this FFQ attempts to minimise error from these challenges by targeting a narrow age range, providing portion size options specifically suited to toddlers and including only those foods of relevance to total and free sugars intakes rather than total diet. To reduce the difficulties arising from the high variability of the toddler diet, participants were asked to report “usual intake”, rather than over a specified period of weeks. However, some participants may have found this more challenging as usual intake may be difficult to define.

The FFQ is well suited to comparing food group contribution to nutrient intake, as further interrogation of FFQ data can be conducted at the line item or block level. However, FFQs are unable to separate out individual sources at the single food level. For example, the SMILE-FFQ is able to identify major sources of sugar by food type (milk, yoghurt, biscuits, chocolate, breakfast cereal, table sugar, etc.), but is unable to describe precisely where, when, and in what combinations these foods are consumed.

### 4.3. Limitations of the Study

Participation in the 24HR may have increased parent awareness of their child’s food intake and influenced their responses to SMILE-FFQ2. This influence could partially explain the wide limits of agreement between repeat administrations of SMILE-FFQ, and why the total sugars results from SMILE-FFQ2 showed greater agreement with 24HR, a finding consistent with other studies of this type [45]. 

It is also likely that social desirability bias played a greater role in the 24HR, which were conducted by a dietitian via telephone interview, than the FFQs, which were self-administered online. This may explain some of the proportional bias; that participants who reported the highest sugars intakes via the online SMILE-FFQ provided more conservative estimates when reporting to the dietitian than those with lower SMILE-FFQ sugars intakes. Other studies support this theory, suggesting social desirability bias influences parents’ reporting of their children’s intakes, particularly in relation to their child’s weight or higher levels of foods that are perceived as unhealthy [46,47]. The recent focus on reducing the intake of free sugars by the scientific community, coupled with current food fads in popular media that demonise sugar intake from any and all sources, may have exacerbated the rates of underreporting of foods high in sugars via telephone interviews. This does not, however, account for the underestimation at lower intakes.

There is some evidence to indicate that seasonal variation may affect dietary assessment responses; however, these effects are generally considered minor, with the greatest variation between different types of fruit and vegetables [48,49]. This FFQ does not differentiate between types of fruit, but rather asks participants to estimate the overall frequency of consumption of any fruit. Additionally, the reference method (24HR) was administered in the middle of the data collection phase, which for most participants (90%), was completed within two months. This is likely to have ameliorated the effect of seasonal change on questionnaire responses.

A further limitation of this study design is the absence of a biomarker for validation. A urinary sugars biomarker based on a 24-h urine collection could be a suitable reference measure in future studies; however, research to date is limited by small sample sizes [50] and a 24-h urine collection in 18–30 month olds may be particularly challenging. In order to provide an estimate of usual intake, multiple collections would be required, resulting in a high cost and subject burden. In the absence of this, there is no reference instrument that can provide an accurate measure of a person’s usual dietary intake. Repeat 24-h recalls are accepted as a suitable but imperfect reference instrument, considered to contain less systematic error than FFQs [11,12]. The collection of intake data on three non-consecutive days in the 24HR may not have been sufficient to represent usual intake for some participants. Use of this imperfect reference instrument may have resulted in correlated errors between the two methods. Conversely, discrepancy between the SMILE-FFQ and 24HR may be the result of departure from true intake by either or both questionnaires. 

### 4.4. Opportunities

As an online tool, the SMILE-FFQ is easily administered and shows potential for use in future Australian dental observational studies involving toddlers and pre-schoolers. If, however, the cohort is substantially different from the one described here, or the tool is modified in any way, an external or internal validation study is required to assess validity [10,11,12]. Further information about the questionnaire is available from the researchers upon request. The SMILE-FFQ has recently been administered to a cohort of approximately 1600 families as part of the NHMRC-funded Study of Mothers and Infants Life Events affecting oral health (SMILE) [15]. 

## 5. Conclusions

The SMILE-FFQ has been developed to capture total and free sugars intake within the unique food landscape of Australian toddlers by parent report. Investigations of relative validity and repeatability suggest that this tool performs similarly to repeat 24-h recalls at ranking participants, with a tendency to underestimate intakes in participants with lower reported 24HR values and overestimate intakes in those with higher reported values. The findings of this study suggest that the SMILE-FFQ is suitable for use in observational studies of Australian toddlers wanting to use ranked total and free sugars intakes. As with all FFQs, it is recommended that future administrations of the tool are accompanied with internal or external calibration or further validity testing if administered to a dissimilar cohort. 

## Figures and Tables

**Table 1:** Demographic characteristics of study participants (*n* = 95).

Characteristics	Mean (SD)
Age of mother (years)	34.2 (±4.4)
Age of child (months)	25 (±4.1)
Gender of child: female (%)	43.2
Mothers’ education: completed university (%)	70.6

**Table 2:** Total and Free sugars as measured by SMILE-FFQ1 ^a^, SMILE-FFQ2, and repeat 24-h recalls.

Method	Mean ± SD	Median	Range	Percentile		Normality			
						Raw		Transformed ^b^	
				25th	75th	Skew	K-S ^c^ *p*	Skew	K-S ^c^ *p*
**Total Sugars:**									
**FFQ1 (g)**	74.8 ± 30.8	66.5	18.9–149.7	51.2	96.3	0.57	0.001	−0.41	0.200
**FFQ2 (g)**	68.7 ± 28.6	65.2	22.6–148.3	49.1	83.6	0.90	0.018	−0.13	0.200
**24HR (g)**	65.9 ± 19.3	64.8	30.0–130.4	52.2	79.0	0.72	0.200	−0.11	0.200
**Free Sugars:**									
**FFQ1 (g)**	22.0 ± 15.5	19.0	1.0–75.6	11.1	26.9	1.31	0.000	−0.63	0.160
**FFQ2 (g)**	21.3 ± 15.0	18.1	1.0–71.8	10.2	29.2	1.26	0.009	−0.93	0.004
**24HR (g)**	18.3 ± 9.7	17.3	2.1–58.7	10.9	24.9	1.05	0.036	−0.72	0.091

^a^ FFQ 1 and 2 refers to the same tool (SMILE-FFQ) administered before (FFQ1) and after (FFQ2) the recalls; **^b^** Natural Log Transformation; **^c^** Kolmogorov–Smirnov test for normality. FFQ: Food Frequency Questionnaire; 24HR: 24-h recalls.

**Table 3:** Relative validity of the SMILE-FFQ ^a^ compared with repeat 24-h recalls.

Method	Correlation	Cross-Classification			
	Pearson ^b^	Chi-Square ^c^			*κ*_w_^d^
		Similarly Classified (%)	Adjacently Classified (%)	Dissimilarly Classified (%)	
**Total Sugars: FFQ1**	0.532 **	54.7	35.8	9.5	0.387
**FFQ2**	0.642 **	55.8	40.0	4.2	0.454
**Free Sugars: FFQ1**	0.392 **	52.6	37.9	9.5	0.359
**FFQ2**	0.484 **	50.4	38.9	10.6	0.320

^a^ FFQ 1 and 2 refers to the same tool (SMILE-FFQ) administered before (FFQ1) and after (FFQ2) the recalls; **^b^** Outcome criteria correlation coefficient (Pearson): Good: ≥0.50; Acceptable: 0.20–0.49; Poor: <0.20 [29,30]; **^c^** Outcome criteria cross-classification (Chi Square): Good: ≥50% similarly classified and ≤10% dissimilarly classified; poor <50% similarly classified and >10% dissimilarly classified [29,30]; **^d^** Outcome criteria Weighted Kappa: good ≥ 0.61; acceptable 0.20–0.6; poor < 0.20 [29,30]. ** *p* < 0.01.

**Table 4:** Bland Altman statistics for the SMILE-FFQ ^a^ compared with repeat 24-h recalls.

Method	Mean Difference ± SD (g)	Agreement *p* Value ^b^	Limits of Agreement LOA (g)	Bland-Altman Index ^c^ (%)
**Total Sugars: FFQ1**	8.9 ± 26.8	0.008	−43.7, 61.4	5.3
**FFQ2**	2.8 ± 22.6	0.596	−41.6, 47.2	5.3
**Free Sugars: FFQ1**	3.7 ± 15.7	0.180	−27.1, 34.5	7.4
**FFQ2**	3.1 ± 15.4	0.490	−27.2, 33.3	7.4

^a^ FFQ 1 and 2 refers to the same tool (SMILE-FFQ) administered before (FFQ1) and after (FFQ2) the recalls; **^b^** Outcome criteria nonparametric agreement (Wilcoxon): Good: *p* > 0.05; Poor: *p* ≤ 0.05 [29]; **^c^** Bland Altman index % of persons outside of LOA. Values ≤ 5% indicate good agreement [31,32].

**Table 5:** Reproducibility of the SMILE-FFQ ^a^.

	Correlation	Cross-Classification			
	Pearson ^b^	Chi-Square ^c^			*κ*_w_^d^
		Similarly Classified (%)	Adjacently Classified (%)	Dissimilarly Classified (%)	
**Total Sugars**	0.494 **	51.5	40.0	8.5	0.359
**Free Sugars**	0.693 **	59.0	34.8	6.3	0.464

^a^ Reproducibility was assessed by comparing results from the SMILE-FFQ administered to the same cohort a minimum of six weeks apart; **^b^** Outcome criteria correlation coefficient (Pearson): Good: ≥0.50; Acceptable: 0.20–0.49; Poor: <0.20 [29,30]; **^c^** Outcome criteria cross-classification (Chi Square): Good: ≥50% similarly classified and ≤10% dissimilarly classified; Poor: <50% similarly classified and >10% dissimilarly classified [29,30]; **^d^** Outcome criteria Weighted Kappa: Good: ≥0.61; Acceptable: 0.20–0.60; Poor: <0.20 [29,30]. ** *p* < 0.01.

**Table 6:** Bland Altman statistics for repeat administrations of the SMILE-FFQ ^a^.

	Mean Difference ± SD (g)	Agreement *p* Value ^b^	Limits of Agreement LOA (g)	Bland-Altman Index ^c^ (%)
**Total Sugars**	6.1 ± 28.5	0.027	−49.8, 61.9	5.3
**Free Sugars**	0.6 ± 12.0	0.583	−22.9, 24.2	6.3

^a^ Reproducibility was assessed by comparing results from the SMILE-FFQ administered to the same cohort a minimum of six weeks apart; **^b^** Outcome criteria nonparametric agreement (Wilcoxon): Good: *p* > 0.05; Poor: *p* ≤ 0.05 [29]; **^c^** Bland Altman index % of persons outside of LOA. Values ≤ 5% indicate good agreement [31,32].

## Supplementary Materials

The following are available online at www.mdpi.com/1660-4601/14/11/1361/s1, Supplementary Material: SMILE Food Frequency Questionnaire.
