# Supplementary material for: Development and Relative Validity of a Food Frequency Questionnaire to Assess Intakes of Total and Free Sugars in Australian Toddlers
Source: Int J Environ Res Public Health. 2017 Nov 8;14(11):1361. doi: 10.3390/ijerph14111361 (PMC5708000; doi:10.3390/ijerph14111361)
Supplement: Supplementary file 1 [file ijerph-14-01361-s001.pdf]

SMILE Food Frequency Questionnaire

INSTRUCTIONS

This questionnaire asks you to describe your child's usual intake of food. Please read and follow these instructions carefully.

This questionnaire:

- is designed to find out what your child ***usually*** eats and drinks, but only for certain food types. It does not ask about every food your child eats.
- looks at current, usual eating habits of your child. We are interested in foods and drinks that are consumed regularly (i.e. more than once per week).
- is meant to collect information about each food ONCE only. Occasionally you may find that some foods could fit into two categories – but please include it in just one.

How to fill in this section

This section asks **how often** your child eats certain foods, and **how much** they eat when they do.

For example, if your child eats plain dried fruit 5 days per week, and when they do they usually eat about 1 tablespoon, fill in the table like this:

Please answer EITHER: This: OR Both of these

My Child Never or Rarely eats this (1)	OR	In a typical week, how often would your child eat the item: 1 time every 2 weeks (2)	1 time per week (3)	2-3 times per week (4)	4-6 times per week (5)	1-2 times per day (6)	3 or more times per day (7)	&	When they eat it, how much would your child usually eat? 4-8 sultanas, 2 dates or dried apricot halves (10g) (1)	1 Tablespoon, 3-4 dates or dried apricot halves (20g) (2)	2 Tablespoons or a snack box (40g) (3)	1/2 cup or 2 snack boxes (80g) (4)	More than 1/2 cup (100g+) (5)
**Dried Fruit: Plain** (2) e.g. sultanas, dried mango, apricot etc. Do NOT include fruit bars or fruit leather													
○	OR	○	○	○	✓	○	○	&	○	✓	○	○	○

Watch out – the amounts change!

And, if your child doesn't eat coated dried fruit, or only very occasionally, fill it in like this:

Please answer EITHER: This: OR Both of these

My Child Never or Rarely eats this (1)	OR	In a typical week, how often would your child eat the item: 1 time every 2 weeks (2)	1 time per week (3)	2-3 times per week (4)	4-6 times per week (5)	1-2 times per day (6)	3 or more times per day (7)	&	When they eat it, how much would your child usually eat? 4-8 sultanas, 2 dates or dried apricot halves (10g) (1)	1 Tablespoon, 3-4 dates or dried apricot halves (20g) (2)	2 Tablespoons or a snack box (40g) (3)	1/2 cup or 2 snack boxes (80g) (4)	More than 1/2 cup (100g+) (5)
**Dried Fruit: Coated** (1) in chocolate, carob, yoghurt etc													
✓	OR	○	○	○	○	○	○	&	○	○	○	○	○

Please note

Please provide your answers for a typical week – rather than what your child ate in the last 7 days, think about their usual intake. Where possible, **include foods your child eats while in the care of others**, for example at childcare or with relatives. Although some toddlers are messy or fussy eaters, **please try to answer based on how much your child actually eats**, rather than what is served to them.

Please try to answer as accurately and honestly as possible. There are no right or wrong answers. You may find it easier to complete this section when you have time to look at foods inside your fridge or pantry!

Milk and Milk Alternatives

In this section:

Milk refers to any animal milk (cow, sheep, goat) in any form (fresh, powdered, long-life etc)

Milk Alternatives refer to any plant based milks (soy, rice, oat, almond etc)

Please answer EITHER: This: OR Both of these

	This:		In a typical week, how often would your child eat the item:							When they eat it, how much would your child usually eat?						
	My Child Never or Rarely eats this (1)	OR	1 time every 2 weeks (2)	1 time per week (3)	2-3 times per week (4)	4-6 times per week (5)	1-2 times per day (6)	3 or more times per day (7)	&	A few sips (less than 50ml) (1)	125mL: Half a cup or baby bottle (2)	200mL: A small cup (eg sippee) or baby bottle (3)	250mL: A medium cup or baby bottle (4)	350mL: A large cup or baby bottle (5)	450mL: A very large cup or baby bottle (6)	600mL: A carton or soft-drink sized bottle (7)
**Flavoured Milk** (1) Includes choc-milk, strawberry milk etc, plus milkshakes and other café milk drinks. Do NOT include plain milk that you add flavour to	○	OR	○	○	○	○	○	○	&	○	○	○	○	○	○	○
**Regular Milk (plain, unflavoured)** (2) Include ALL plain milk consumed, including milk given as a drink (with or without additions), added in cooking or when served	○	OR	○	○	○	○	○	○	&	○	○	○	○	○	○	○
**Flavoured Milk Alternatives** (3) Includes soy and other alternative milk products purchased flavoured with chocolate, strawberry etc, plus milkshakes and other café items ordered with milk alternatives	○	OR	○	○	○	○	○	○	&	○	○	○	○	○	○	○
**Regular Milk Alternatives (plain, unflavoured)** (4) Include ALL plain milk alternatives consumed, including milk given as a drink, added in cooking or when served	○	OR	○	○	○	○	○	○	&	○	○	○	○	○	○	○
**Infant Formula and Toddler Milk (all types)** (5) Include ALL formulations for infants or toddlers, including regular step 1-4 powders, regular and special formulations, made from milk or milk alternatives.	○	OR	○	○	○	○	○	○	&	○	○	○	○	○	○	○

Condensed and Evaporated Milk

Please answer EITHER:

This: | OR Both of these

My Child Never or Rarely eats this (1)	OR	In a typical week, how often would your child eat the item:						&	When they eat it, how much would your child usually eat?									
		1 time every 2 weeks (2)	1 time per week (3)	2-3 times per week (4)	4-6 times per week (5)	1-2 times per day (6)	3 or more times per day (7)	&	1 teaspoons (5mL) (1)	2 teaspoons (10mL) (2)	3 teaspoons (15mL) (3)	1 Tablespoons (20mL) (4)	2 Tablespoons (40mL) (5)	3 Tablespoons (60mL) (6)	1/4 Tin (100mL) (7)	1/2 Tin (200mL) (8)	3/4 Tin (300mL) (9)	1 or more tins (400mL+) (10)
**Condensed Milk** (1)																		
○	OR	○	○	○	○	○	○	&	○	○	○	○	○	○	○	○	○	○
**Evaporated Milk** (2)																		
○	OR	○	○	○	○	○	○	&	○	○	○	○	○	○	○	○	○	○

Cheese

Please answer EITHER:

This: | OR Both of these

My Child Never or Rarely eats this (1)	OR	In a typical week, how often would your child eat the item:						&	When they eat it, how much would your child usually eat?						
		1 time every 2 weeks (2)	1 time per week (3)	2-3 times per week (4)	4-6 times per week (5)	1-2 times per day (6)	3 or more times per day (7)	&	1 small square or smear (enough for 1 cracker) (1)	20g: 1 single serve portion, slice or 2 (1cm) cubes (2)	40g: 2 slices or 4 (1cm) cubes (3)	70g: 3-4 slices or half a cup, grated (4)	100g: 5 slices or 3/4 of a cup, grated (5)	120g: 6 slices or 1 cup, grated (6)	150g+: More than 6 slices (7)
**Cream Cheese or Ricotta** (1) Include individually wrapped cream cheese such as Laughing Cow															
○	OR	○	○	○	○	○	○	&	○	○	○	○	○	○	○
**Other Soft Cheeses** (2) Including feta, brie etc															
○	OR	○	○	○	○	○	○	&	○	○	○	○	○	○	○
**Hard Cheese** (3) Include cheddar, tasty, mozzarella, grated cheese blends, cheese sticks, single wrapped slices etc															
○	OR	○	○	○	○	○	○	&	○	○	○	○	○	○	○
**Cheese Alternatives: Any Type** (4) Include any kind of hard or soft cheese alternative, Notzarella, Tofutti, soy cheese etc															
○	OR	○	○	○	○	○	○	&	○	○	○	○	○	○	○

Yoghurt

Please answer EITHER:

This: / OR Both of these

Item	My Child Never or Rarely eats this (1)	OR	In a typical week, how often would your child eat the item: 1 time every 2 weeks (2)	1 time per week (3)	2-3 times per week (4)	4-6 times per week (5)	1-2 times per day (6)	3 or more times per day (7)	&	When they eat it, how much would your child usually eat? 3 teaspoons (20mL or less) (1)	2-3 Tablespoons, a small pouch (60mL) (2)	Less than 1/2 cup, or 1 junior tub or regular pouch (100mL) (3)	More than 1/2 cup, or a medium tub (150mL) (4)	2/3 cup, a regular tub (adult tub) (200mL) (5)	1 cup, more than a regular tub (250mL+) (6)
**Children's Yoghurt** (1)<br>Anything with a cartoon character on the package, &/or yoghurt sold in a mini tub, pouch, stick etc	○	OR	○	○	○	○	○	○	&	○	○	○	○	○	○
**Plain or Natural Yoghurt** (2)<br>Anything that is not flavoured	○	OR	○	○	○	○	○	○	&	○	○	○	○	○	○
**Fruit Flavoured Yoghurt** (3)<br>e.g. strawberry, banana, fruits of the forest, coconut, apricot etc	○	OR	○	○	○	○	○	○	&	○	○	○	○	○	○
**Any Other Flavoured Yoghurt** (4)<br>e.g. vanilla, honey, French cheesecake etc	○	OR	○	○	○	○	○	○	&	○	○	○	○	○	○
**Probiotic Yoghurt Drinks** (5)<br>e.g. Yakult, Vaalia Innergy, Coles Pro-B etc	○	OR	○	○	○	○	○	○	&	○	○	○	○	○	○
**Yoghurt Alternatives: Flavoured** (6)<br>Yoghurt made from soy, coconut or other plant-based milk alternatives; include fruit flavours and dessert styles, such as mixed berry, mango, vanilla etc	○	OR	○	○	○	○	○	○	&	○	○	○	○	○	○
**Yoghurt Alternatives: Plain** (7)<br>Yoghurt made from soy, coconut or other plant-based milk alternatives; include any plain, natural or unflavoured types	○	OR	○	○	○	○	○	○	&	○	○	○	○	○	○

If your child eats yoghurt, do you choose reduced fat versions?

- ❍ Usually (1)
- ❍ Sometimes (2)
- ❍ Never or Rarely (3)
- ❍ I don't know or my child does not eat yoghurt (4)

Cream and Custard

Please answer EITHER: This: OR Both of these

	My Child Never or Rarely eats this (1)	OR	In a typical week, how often would your child eat the item: 1 time every 2 weeks (2)	1 time per week (3)	2-3 times per week (4)	4-6 times per week (5)	1-2 times per day (6)	3 or more times per day (7)	&	When they eat it, how much would your child usually eat? 3 teaspoons (20mL or less) (1)	2-3 Tablespoons, a small pouch (60mL) (2)	1/2 cup, a junior tub or regular pouch (125mL) (3)	2/3 cup (200mL) (4)	1 cup or more (250mL+) (5)
**Cream or Sour Cream** (1) Unsweetened varieties only	❍	OR	❍	❍	❍	❍	❍	❍	&	❍	❍	❍	❍	❍
**Infant and Toddler Custard: Plain or Vanilla Only** (2) Usually found in the baby aisle, or in single-serve pouches in the fridge section	❍	OR	❍	❍	❍	❍	❍	❍	&	❍	❍	❍	❍	❍
**Regular Custard: Plain or Vanilla Only** (3) Include store-bought or home-made custards	❍	OR	❍	❍	❍	❍	❍	❍	&	❍	❍	❍	❍	❍
**All Other Kinds of Flavoured Custard and Custard-like Desserts** (4) e.g. YoGo, SnakPack, any chocolate or banana custard, sweetened or sweet-flavoured cream, chocolate mousse, baked custard etc	❍	OR	❍	❍	❍	❍	❍	❍	&	❍	❍	❍	❍	❍
**Other milk-based desserts (not frozen)** (5) Such as rice pudding (store-bought or home-made)	❍	OR	❍	❍	❍	❍	❍	❍	&	❍	❍	❍	❍	❍

Frozen Desserts

In this section: Consider those purchased at the supermarket, as well as from a deli, petrol station, ice cream shop, drive-thru, grandma's house etc

Please answer EITHER: This: OR Both of these

My Child Never or Rarely eats this (1)	OR	In a typical week, how often would your child eat the item:						&	When they eat it, how much would your child usually eat?				
		1 time every 2 weeks (2)	1 time per week (3)	2-3 times per week (4)	4-6 times per week (5)	1-2 times per day (6)	3 or more times per day (7)		3 teaspoons (20g or less) (1)	1 small scoop or mini-pop (around 40g) (2)	1 medium scoop or medium serve on a stick eg a Choc Wedge (80g) (3)	2 medium scoops or a large serve on a stick eg a Magnum (125g) (4)	3 scoops or more (175g+) (5)
**Ice cream and frozen yoghurt** (1)<br>Includes any frozen, milk-based dessert or treat, in a tub, on a stick, bought by the scoop etc													
○	OR	○	○	○	○	○	○	&	○	○	○	○	○
**Icy Poles and Sorbet** (2)<br>Includes any other frozen desserts and treats that are like ice cream, but NOT made from milk or milk alternatives													
○	OR	○	○	○	○	○	○	&	○	○	○	○	○
**Ice Cream Alternatives and Other Frozen Milk Alternatives** (3)<br>Includes any dairy free, milk-style frozen desserts such as soy ice cream in a tub or on a stick, frozen soy or coconut yoghurt, Tofutti Cuties etc													
○	OR	○	○	○	○	○	○	&	○	○	○	○	○

Fruit

Please answer EITHER: This: OR Both of these

My Child Never or Rarely eats this (1)	OR	In a typical week, how often would your child eat the item:						&	When they eat it, how much would your child usually eat?			
		1 time every 2 weeks (2)	1 time per week (3)	2-3 times per week (4)	4-6 times per week (5)	1-2 times per day (6)	3 or more times per day (7)		30g: eg 1/2 a plum, mandarin or kiwi, 1 small apricot, or 4 grapes or large berries (1)	70g: eg 1 small piece (plum, kiwi etc), 6-8 grapes or large berries, 1/2 a medium piece (apple, banana, orange etc) (2,3)	150g: eg 1 medium piece (apple, banana, pear, orange), 2 small pieces, or 1 cup of grapes or berries (4)	175g+: More than 1 medium piece or 2 small pieces (5)
**Fresh or Frozen Fruit: All Types** (1)<br>e.g. banana, apple, watermelon, grapes, pear, berries etc												
○	OR	○	○	○	○	○	○	&	○	○	○	○

Dried Fruit

Please answer EITHER:

This: | OR Both of these

My Child Never or Rarely eats this (1)	OR	In a typical week, how often would your child eat the item: 1 time every 2 weeks (2)	1 time per week (3)	2-3 times per week (4)	4-6 times per week (5)	1-2 times per day (6)	3 or more times per day (7)	&	When they eat it, how much would your child usually eat? 4-8 sultanas, 2 dates or dried apricot halves (10g) (1)	1 Tablespoon, 3-4 dates or dried apricot halves (20g) (2)	2 Tablespoons or a snack box (40g) (3)	1/2 cup or 2 snack boxes (80g) (4)	More than 1/2 cup (100g+) (5)

Dried Fruit: Coated (1) in chocolate, carob, yoghurt etc

❍	OR	❍	❍	❍	❍	❍	❍	&	❍	❍	❍	❍	❍

Dried Fruit: Plain (2) e.g. sultana, dried mango, apricot etc. Do NOT include fruit bars or fruit leather

❍	OR	❍	❍	❍	❍	❍	❍	&	❍	❍	❍	❍	❍

Tinned Fruit

Please answer EITHER:

This: | OR Both of these

My Child Never or Rarely eats this (1)	OR	In a typical week, how often would your child eat the item: 1 time every 2 weeks (2)	1 time per week (3)	2-3 times per week (4)	4-6 times per week (5)	1-2 times per day (6)	3 or more times per day (7)	&	When they eat it, how much would your child usually eat? 20g: eg 1 peach slice or apricot half (1)	40g: eg 2 apricot halves or 1 pineapple ring (2)	80g: eg 4 peach slices, 4 apricot halves, 1 pear half (3)	120g: eg 1/2 cup or 1 snack tub (4)	160g: eg 8 peach slices, 2 pear halves (5)	200g: eg 3/4 cup, 1/2 a regular tin (6)	240g: eg 1 cup, 3 pear halves (7)

Tinned Fruit: Whole or Pieces (1) not puree

❍	OR	❍	❍	❍	❍	❍	❍	&	❍	❍	❍	❍	❍	❍	❍

If your child eats tinned fruit, which do you usually choose?

- ❍ Fruit in syrup (1)
- ❍ Fruit in natural juice (2)
- ❍ Both types equally (3)
- ❍ I don't know or my child does not eat tinned fruit (4)

If your child eats tinned fruit, do they eat or drink the syrup or juice?

- ❍ Usually (1)
- ❍ Sometimes (2)
- ❍ Never or Rarely (3)
- ❍ I don't know or my child does not eat tinned fruit (4)

Pureed Fruit and Vegetables

Please answer EITHER: This: OR Both of these

	This:	OR	In a typical week, how often would your child eat the item:						&	When they eat it, how much would your child usually eat?					
	My Child Never or Rarely eats this (1)		1 time every 2 weeks (2)	1 time per week (3)	2-3 times per week (4)	4-6 times per week (5)	1-2 times per day (6)	3 or more times per day (7)		2 teaspoons (15g or less) (1)	1-2 Tablespoons (35g) (2)	3 Tablespoons or 1/2 a pouch or baby jar (65g) (3)	1/2 cup or 1 pouch or baby jar (120g) (4)	3/4 cup, 1.5 pouches (200g) (5)	1 cup, 2 pouches or baby jars or more (250g+) (6)
**Toddler or Infant Fruit Puree** (1)<br>Usually found in the baby aisle: sold in jars, pouches, tins etc		OR							&						
**Toddler or Infant Vegetable Puree** (2)<br>Usually found in the baby aisle: sold in jars, pouches, tins etc.		OR							&						
**Fruit Puree: Other, Shop Bought** (3)<br>Not from the baby aisle		OR							&						
**Fruit Puree: Home-Made** (4)		OR							&						

Nuts

Please answer EITHER: This: OR Both of these

Item	My Child Never or Rarely eats this (1)	OR	1 time every 2 weeks (2)	1 time per week (3)	2-3 times per week (4)	4-6 times per week (5)	1-2 times per day (6)	3 or more times per day (7)	&	5 nuts or less (5-10g) (1)	10 nuts or less (15-25g) (2)	1-2 Tablespoons, 15-20 small nuts (3)	1/4 cup (35-50g) (4)	1/2 cup (75-100g) (5)	3/4 cup or more (120g+) (6)
			In a typical week, how often would your child eat the item:							When they eat it, how much would your child usually eat?					
**Nuts: Coated** (1) in chocolate, carob, yoghurt, honey etc	❍	OR	❍	❍	❍	❍	❍	❍	&	❍	❍	❍	❍	❍	❍
**Nuts: All Other Types** (2) Include roasted or raw, whole or chopped, salted or spiced etc, Do NOT include nut pastes	❍	OR	❍	❍	❍	❍	❍	❍	&	❍	❍	❍	❍	❍	❍

Nut Pastes and Other Spreads

Please answer EITHER: This: OR Both of these

Item	My Child Never or Rarely eats this (1)	OR	1 time every 2 weeks (2)	1 time per week (3)	2-3 times per week (4)	4-6 times per week (5)	1-2 times per day (6)	3 or more times per day (7)	&	teaspoons 1/2 (1)	teaspoons 1 (2)	teaspoons 2 (3)	teaspoons 3 (4)	Tablespoons 1 (5)	Tablespoons 2 (6)	Tablespoons 3 (7)	Tablespoons 4 or more (8)
			In a typical week, how often would your child eat the item:							When they eat it, how much would your child usually eat?							
**Nut Paste** (1) Include peanut butter, almond spread, cashew paste etc. Do NOT include chocolate flavoured	❍	OR	❍	❍	❍	❍	❍	❍	&	❍	❍	❍	❍	❍	❍	❍	❍
**Chocolate or Carob Spread** (2) With or without nuts e.g. Nutella, Kraft Hazelnut spread, Sweet William Chocolate etc	❍	OR	❍	❍	❍	❍	❍	❍	&	❍	❍	❍	❍	❍	❍	❍	❍
**Jam, Marmalade and Other Fruit Spreads** (3) Store-bought or home-made	❍	OR	❍	❍	❍	❍	❍	❍	&	❍	❍	❍	❍	❍	❍	❍	❍
**Honey** (4)	❍	OR	❍	❍	❍	❍	❍	❍	&	❍	❍	❍	❍	❍	❍	❍	❍

Breakfast Cereal

Please answer EITHER: This: OR Both of these

	My Child Never or Rarely eats this (1)	OR	In a typical week, how often would your child eat the item: 1 time every 2 weeks (2)	1 time per week (3)	2-3 times per week (4)	4-6 times per week (5)	1-2 times per day (6)	3 or more times per day (7)	&	When they eat it, how much would your child usually eat? A small sprinkle, 1-2 Tablespoons or 1/2 a wheat biscuit (1)	1/4 cup (30g muesli, 15g flakes) or 1 wheat biscuit (2)	1/2 cup (60g muesli, 25g flakes) or 2 wheat biscuits (3)	1 cup (120g muesli, 50g flakes) or 3 wheat biscuits (4)	More than 1 cup, 4 or more wheat biscuits (5)
**Porridge or Other Hot Cereal** (1)	❍	OR	❍	❍	❍	❍	❍	❍	&	❍	❍	❍	❍	❍
**Wheat Biscuits: Plain** (2) e.g. Weet-Bix, Vita Brits etc	❍	OR	❍	❍	❍	❍	❍	❍	&	❍	❍	❍	❍	❍
**Puffed, Unflavoured Cereal** (3) e.g. puffed corn, puffed rice	❍	OR	❍	❍	❍	❍	❍	❍	&	❍	❍	❍	❍	❍
**Muesli: Toasted** (4)	❍	OR	❍	❍	❍	❍	❍	❍	&	❍	❍	❍	❍	❍
**Muesli: Untoasted** (5)	❍	OR	❍	❍	❍	❍	❍	❍	&	❍	❍	❍	❍	❍
**Cereal Flakes** (6) e.g. Cornflakes, Weeties, Sultana Bran, Light n Tasty etc	❍	OR	❍	❍	❍	❍	❍	❍	&	❍	❍	❍	❍	❍
**Flavoured Breakfast Cereal: All Other Types** (7) e.g. Coco-Pops, Honey Puffs, Cheerios, Nutri-Grain, MiLo, Mini-Wheats, Weet-Bix Bites etc	❍	OR	❍	❍	❍	❍	❍	❍	&	❍	❍	❍	❍	❍

Biscuits

In this section: Include both **home-made and commercial** varieties

Please answer EITHER:

This: / **OR Both of these**

My Child Never or Rarely eats this (1)	OR	In a typical week, how often would your child eat the item:						&	When they eat it, how much would your child usually eat?					
		1 time every 2 weeks (2)	1 time per week (3)	2-3 times per week (4)	4-6 times per week (5)	1-2 times per day (6)	3 or more times per day (7)	&	1-2 bites or mini-biscuits, or 1 mini meringue (1)	1/2 a medium biscuit or 4 mini-biscuits (2)	1 medium biscuit or meringue (3)	2 medium biscuits, or 1 snack pack (4)	3 medium biscuits (5)	4 or more medium biscuits (6)

Plain, Sweet Biscuits and Wafers (1)

e.g. Milk Arrowroot, Tiny Teddy, Tina Wafer, Scotch Finger (uncoated), Wheatmeal, Animal Biscuit, Vanilla bite, Shortbread etc. Include shop-bought and home-made.

❍	OR	❍	❍	❍	❍	❍	❍	&	❍	❍	❍	❍	❍	❍

All Other Sweet Biscuits and Cookies (2)

Include filled or coated varieties e.g. Choc-chip, Tim-Tam, Tic-Toc, Monte Carlo, Jam Drop, Melting Moment, Full-o-Fruit etc. Include shop-bought and home-made.

❍	OR	❍	❍	❍	❍	❍	❍	&	❍	❍	❍	❍	❍	❍

Meringue or Honeycomb (3)

❍	OR	❍	❍	❍	❍	❍	❍	&	❍	❍	❍	❍	❍	❍

Bars and Bar-Like Snack Food

In this section: Include both home-made and commercial varieties

Please answer EITHER:

This: | OR Both of these

My Child Never or Rarely eats this (1)	OR	In a typical week, how often would your child eat the item: 1 time every 2 weeks (2)	1 time per week (3)	2-3 times per week (4)	4-6 times per week (5)	1-2 times per day (6)	3 or more times per day (7)	&	When they eat it, how much would your child usually eat? 15g: eg 1/2 a medium bar, 1 Roll-Up (1)	20g: eg 1 fruit stick, 1 small crackle, 1 LCM (2)	30g: eg 1 medium bar, 2 roll ups (3)	40g: eg 2 fruit sticks, 1 large or 2 small crackles (4)	50g: eg 1 large bar, 3 small crackles (5)	60g: eg 2 medium bars (6)	100g: eg 1 extra large bar (7)
**Honey Joys, Chocolate Crackles and Puffed Cereal Bars (1)** e.g. LCM Nutri-Grain bar, Milo bar, honey crackle etc															
○	OR	○	○	○	○	○	○	&	○	○	○	○	○	○	○
**Cake or Muffin Bars and Muesli Bars (2)** e.g. Yoghurt tops, K-Time twist, breakfast bar, chewy/crunchy muesli bar, trail bar, nut bar, oat bar etc															
○	OR	○	○	○	○	○	○	&	○	○	○	○	○	○	○
**Fruit Flavoured Snacks (3)** includes fruit sticks, straps, balls, squares, bars, leather, Roll-Ups etc															
○	OR	○	○	○	○	○	○	&	○	○	○	○	○	○	○

Cakes and Puddings

In this section: Include both home-made and commercial varieties

Please answer EITHER:

This: | OR Both of these

My Child Never or Rarely eats this (1)	OR	In a typical week, how often would your child eat the item: 1 time every 2 weeks (2)	1 time per week (3)	2-3 times per week (4)	4-6 times per week (5)	1-2 times per day (6)	3 or more times per day (7)	&	When they eat it, how much would your child usually eat? 1/2 a cupcake, 1 medium doughnut or 2 mini muffins (40g) (1)	1 cupcake, large doughnut or small muffin (80g) (2)	1 medium café muffin (120g) (3)	2 cupcakes, 4 doughnuts or a large café muffin (160g) (4)
**Cakes, Muffins, Slices, Doughnuts and Puddings (1)** Includes brownies, cup-cakes, iced or plain doughnuts, cakes and cake-based desserts such as self-saucing pudding, fruit cake, bread pudding, lemon delicious etc												
○	OR	○	○	○	○	○	○	&	○	○	○	○

Sweet Bread and Pastry

In this section: Include both home-made and commercial varieties

Please answer EITHER: This: OR Both of these

Item	My Child Never or Rarely eats this (1)	OR	In a typical week, how often would your child eat the item:						&	When they eat it, how much would your child usually eat?					
			1 time every 2 weeks (2)	1 time per week (3)	2-3 times per week (4)	4-6 times per week (5)	1-2 times per day (6)	3 or more times per day (7)		1/2 a pancake or 1 large pikelet (40g) (1)	1/2 a medium croissant or finger bun, or 1 slice of fruit bread (50g) (2, 3)	1 pancake, 2-3 pikelets (80g) (4)	1 medium croissant or finger bun, or 2 slices of fruit bread (100g) (5, 6)	2 pancakes, or 4-6 pikelets (160g) (7)	More than 3 pancakes or 8 pikelets (200g+) (8)
**Sweet Bread and Pancakes** (1)<br>Includes finger bun, fruit bread, cinnamon scroll, pikelet, scone, waffle etc.	○	OR	○	○	○	○	○	○	&	○	○	○	○	○	○
**Sweet Pastry** (2)<br>Includes croissant, strudel, Danish pastry, baklava etc	○	OR	○	○	○	○	○	○	&	○	○	○	○	○	○

Savoury Snack Food

Please answer EITHER: This: OR Both of these

Item	My Child Never or Rarely eats this (1)	OR	In a typical week, how often would your child eat the item:						&	When they eat it, how much would your child usually eat?							
			1 time every 2 weeks (2)	1 time per week (3)	2-3 times per week (4)	4-6 times per week (5)	1-2 times per day (6)	3 or more times per day (7)		10g: eg 6 rice crackers or Shapes, 2 Jatz, 1 Sao or Corn Thin (1)	15g: eg 2 Cruskits or 1 thick rice cake (2)	25g: eg 1 snack pack, 10 crisps or corn chips, 1 'row' of rice crackers (3)	40g: eg 4 corn thins, 5 Cheds, 8 Jatz (4)	50g: eg 2 rows of rice crackers, 5 Sao, 1/4 of a large packet of Chips or box of Shapes etc (5, 6)	100g: eg 1/2 a large packet of crisps, or box of shapes etc or 1 packet of rice crackers (7)	120g+: more than 1/2 a large packet or box (8)	150g+: More than 6 slices (8)
**Plain Savoury Biscuits or Crackers** (1)<br>e.g. Sao, Jatz, water thins, plain rice crackers, plain corn thins, plain rice cakes, plain breadsticks etc	○	OR	○	○	○	○	○	○	&	○	○	○	○	○	○	○	○
**Flavoured Savoury Biscuits or Crackers** (2)<br>e.g. Chedz, Shapes, flavoured rice crackers, flavoured corn cakes or rice thins etc	○	OR	○	○	○	○	○	○	&	○	○	○	○	○	○	○	○
**Savoury Snack Foods** (3)<br>Includes potato crisps, corn chips, prawn crackers, Rice Wheels, Mamee noodle snack, Cheese Fiddlesticks etc	○	OR	○	○	○	○	○	○	&	○	○	○	○	○	○	○	○

Drinks

In this section: Some of these may be difficult to answer if your child sips throughout the day. If that is the case, try to get the total amount correct.
For example, if your child had 200ml of juice in sips over the whole day, you could report 50ml three or more times a day, or 125ml one to two times per day.

Please answer EITHER: **This:** **OR Both of these**

Item	My Child Never or Rarely eats this (1)	OR	In a typical week, how often would your child eat the item: 1 time every 2 weeks (2)	1 time per week (3)	2-3 times per week (4)	4-6 times per week (5)	1-2 times per day (6)	3 or more times per day (7)	&	When they eat it, how much would your child usually eat? A few sips (50ml or less) (1)	125mL: Half a cup or baby bottle or 1 mini juice box (2)	200mL: A small cup (eg sippee), baby bottle or mini can (3)	250mL: A medium cup, baby bottle or regular juice box (4)	350mL: A large cup or regular can (5)	450mL: A very large cup or medium drink bottle (6)	600mL: A soft-drink sized bottle (7)	More than 600mL (6)
**Water: Plain, Still** (1)<br>Tap, bottled etc	○	OR	○	○	○	○	○	○	&	○	○	○	○	○	○	○	○
**Water: Plain, Carbonated** (2)<br>e.g. soda water, sparkling water etc	○	OR	○	○	○	○	○	○	&	○	○	○	○	○	○	○	○
**Water: Lightly Flavoured, Clear** (3)<br>Although there is some flavour, these water-based drinks are still clear, e.g. PumP water with a twist, PLaY fruit water, coconut water etc	○	OR	○	○	○	○	○	○	&	○	○	○	○	○	○	○	○
**Junior Juice** (4)<br>Infant or toddler juice, usually found in the baby aisle	○	OR	○	○	○	○	○	○	&	○	○	○	○	○	○	○	○
**All Other Fruit Juice or Fruit Juice Drinks** (5)<br>Including poppers, juice boxes and bottled juice sold refrigerated or at room temperature, purchased in a café, home squeezed etc	○	OR	○	○	○	○	○	○	&	○	○	○	○	○	○	○	○
**Vegetable Juice** (6)<br>Include store bought or home-made	○	OR	○	○	○	○	○	○	&	○	○	○	○	○	○	○	○

Continued over

Drinks *continued*

Please answer EITHER: **This:** ... **OR Both of these**

Item	My Child Never or Rarely eats this (1)	OR	In a typical week, how often would your child eat the item: 1 time every 2 weeks (2)	1 time per week (3)	2-3 times per week (4)	4-6 times per week (5)	1-2 times per day (6)	3 or more times per day (7)	&	When they eat it, how much would your child usually eat? A few sips (50ml or less) (1)	125mL: Half a cup or baby bottle or 1 mini juice box (2)	200mL: A small cup (eg sippee), baby bottle or mini can (3)	250mL: A medium cup, baby bottle or regular juice box (4)	350mL: A large cup or regular can (5)	450mL: A very large cup or medium drink bottle (6)	600mL: A soft-drink sized bottle (7)	More than 600mL (6)
**Cordial: Diet, Lite or Sugarfree** (7)	○	OR	○	○	○	○	○	○	&	○	○	○	○	○	○	○	○
**Cordial: All Other Types** (8)	○	OR	○	○	○	○	○	○	&	○	○	○	○	○	○	○	○
**Soft Drink: Diet, Lite or Sugarfree** (9)	○	OR	○	○	○	○	○	○	&	○	○	○	○	○	○	○	○
**Soft Drink: All Other Types** (10) Include cola, fruit flavours, energy drinks, red creaming soda, lemonade etc	○	OR	○	○	○	○	○	○	&	○	○	○	○	○	○	○	○
**Frozen Drinks** (11) e.g. slushy, granita, Frozen Coke etc	○	OR	○	○	○	○	○	○	&	○	○	○	○	○	○	○	○
**Tea: From Leaves** (12) Any types from loose leaf or tea bag, including black, white, green, herbal, tisane, chai leaf etc	○	OR	○	○	○	○	○	○	&	○	○	○	○	○	○	○	○
**Tea: From Powder, Syrup or Pre-made** (13) e.g. chai powder or syrup, Lipton iced tea etc	○	OR	○	○	○	○	○	○	&	○	○	○	○	○	○	○	○

If your child drinks juice, do you look for products with "no added sugar" or "100% fruit juice" on the label?

- ❍ Usually (1)
- ❍ Sometimes (2)
- ❍ Never or Rarely (3)
- ❍ I don't know or my child does not drink juice (4)

Drink Powder

Please answer EITHER: This: OR Both of these

My Child Never or Rarely eats this (1)	OR	In a typical week, how often would your child eat the item: 1 time every 2 weeks (2)	1 time per week (3)	2-3 times per week (4)	4-6 times per week (5)	1-2 times per day (6)	3 or more times per day (7)	&	When they eat it, how much would your child usually eat? teaspoons 1/2 (1)	teaspoons 1 (2)	teaspoons 2 (3)	teaspoons 3 (4)	Tablespoons 1 (5)	Tablespoons 2 (6)	Tablespoons 3 (7)	Tablespoons 4 or more (8)
**Drink Powder: Added Vitamins and Minerals** (1)<br>e.g. Milo, Ovaltine, Sustagen etc																
❍	OR	❍	❍	❍	❍	❍	❍	&	❍	❍	❍	❍	❍	❍	❍	❍
**Drink Powder: Regular** (2)<br>e.g. drinking chocolate, Nesquik (any flavour), Sipahh straws etc																
❍	OR	❍	❍	❍	❍	❍	❍	&	❍	❍	❍	❍	❍	❍	❍	❍

What does your child usually drink from?

	My child rarely or never uses this(1)	My child sometimes uses this(2)	My child regularly or always uses this(3)
A regular cup or glass without a straw(1)	❍	❍	❍
A regular cup or glass with a straw(2)	❍	❍	❍
A sipper cup(3)	❍	❍	❍
A bottle with a teat(4)	❍	❍	❍
A sports or drink bottle with straw, pop-top etc(5)	❍	❍	❍
Other, please give details(6):	❍	❍	❍

Sauces and Condiments

Please answer EITHER: This: OR Both of these

	My Child Never or Rarely eats this (1)	OR	In a typical week, how often would your child eat the item: 1 time every 2 weeks (2)	1 time per week (3)	2-3 times per week (4)	4-6 times per week (5)	1-2 times per day (6)	3 or more times per day (7)	&	When they eat it, how much would your child usually eat? teaspoons 1/2 (1)	teaspoons 1 (2)	teaspoons 2 (3)	teaspoons 3 (4)	Tablespoons 1 (5)	Tablespoons 2 (6)	Tablespoons 3 (7)	Tablespoons 4 or more (8)
**Tomato or Barbecue Sauce** (1) Also known as ketchup	❍	OR	❍	❍	❍	❍	❍	❍	&	❍	❍	❍	❍	❍	❍	❍	❍
**Sweet Marinades and Sauces** (2) Such as oyster sauce, kecap manis, sweet chilli, hoisin, plum sauce etc	❍	OR	❍	❍	❍	❍	❍	❍	&	❍	❍	❍	❍	❍	❍	❍	❍
**Mayonnaise: Low Fat** (3) Include all reduced fat types e.g. traditional, egg mayo, aioli etc	❍	OR	❍	❍	❍	❍	❍	❍	&	❍	❍	❍	❍	❍	❍	❍	❍
**Mayonnaise: Not Low Fat** (4) Include all regular fat types e.g. traditional, egg mayo, aioli etc	❍	OR	❍	❍	❍	❍	❍	❍	&	❍	❍	❍	❍	❍	❍	❍	❍
**Chutney or Relish** (5) e.g. mango chutney, corn relish, tomato chutney, onion relish etc	❍	OR	❍	❍	❍	❍	❍	❍	&	❍	❍	❍	❍	❍	❍	❍	❍
**Dessert Toppings** (6) e.g. chocolate, caramel, butterscotch, strawberry sauces, Ice Magic etc	❍	OR	❍	❍	❍	❍	❍	❍	&	❍	❍	❍	❍	❍	❍	❍	❍

Sugar and Sugar Substitutes

In this section: Include sugar added in cooking or serving, sprinkled on cereal, stirred into a drink etc. Do NOT include sugar used to bake cakes or biscuits.

Please answer EITHER:

This: **OR Both of these**

My Child Never or Rarely eats this (1)	OR	In a typical week, how often would your child eat the item:						&	When they eat it, how much would your child usually eat?							
		1 time every 2 weeks (2)	1 time per week (3)	2-3 times per week (4)	4-6 times per week (5)	1-2 times per day (6)	3 or more times per day (7)		1/2 (1) teaspoons	1 (2) teaspoons	2 (3) teaspoons	3 (4) teaspoons	1 (5) Tablespoons	2 (6) Tablespoons	3 (7) Tablespoons	4 or more (8) Tablespoons

Sugar: Solid or Granulated (1)

Include regular table sugar, white, brown, raw, palm and date sugar

❍	OR	❍	❍	❍	❍	❍	❍	&	❍	❍	❍	❍	❍	❍	❍	❍

Sugar: Syrups Other Than Honey (2)

Such as agave, maple, golden syrup, corn or rice syrup

❍	OR	❍	❍	❍	❍	❍	❍	&	❍	❍	❍	❍	❍	❍	❍	❍

Sweeteners: No or Low Calorie (3)

e.g. Equal, Splenda, Stevia, Nutrasweet etc

❍	OR	❍	❍	❍	❍	❍	❍	&	❍	❍	❍	❍	❍	❍	❍	❍

Jelly

Please answer EITHER:

This: **OR Both of these**

My Child Never or Rarely eats this (1)	OR	In a typical week, how often would your child eat the item:						&	When they eat it, how much would your child usually eat?					
		1 time every 2 weeks (2)	1 time per week (3)	2-3 times per week (4)	4-6 times per week (5)	1-2 times per day (6)	3 or more times per day (7)		2 teaspoons (10mL) (1)	2 Tablespoons (40mL) (2)	1/4 of a cup (60mL) (3)	1/2 a cup (125mL) (4)	1 cup (250mL) (5)	More than 1 cup (6)

Jelly: Diet, Lite or Sugarfree (1)

Do NOT include "natural" or "25% less sugar" jelly in this group

Note: 1 standard box of jelly crystals makes 2 cups (500ml) of jelly

❍	OR	❍	❍	❍	❍	❍	❍	&	❍	❍	❍	❍	❍	❍

Jelly: Regular (2)

Include all other types, such as natural, 25% less sugar, agar jelly, fruit flavours, make-your-own flavour etc

❍	OR	❍	❍	❍	❍	❍	❍	&	❍	❍	❍	❍	❍	❍

Chocolate and Lollies

Note: lollies are also known as candy or sweets

Please answer EITHER: This: OR Both of these

Item	My Child Never or Rarely eats this (1)	OR	In a typical week, how often would your child eat the item: 1 time every 2 weeks (2)	1 time per week (3)	2-3 times per week (4)	4-6 times per week (5)	1-2 times per day (6)	3 or more times per day (7)	&	When they eat it, how much would your child usually eat? 10g: eg 2 soft or hard lollies, 2 squares of chocolate, 1 Redskin or lollipop, 2 marshmallows (1,2)	20g: eg 1 fun-size bar, 4 lollies (3)	25g: eg 5 lollies or marshmallows, 1 row of chocolate (4)	40g: eg 2 fun-size bars, 8 lollies (5)	50g: eg 10 lollies or marshmallows, 2 rows of chocolate, 1 regular chocolate bar, 1/3 of a bag of lollies (6,7)	75g: eg 1 large chocolate bar (king size), 1/2 a bag of lollies (8)	100g or more (9)
**Chocolate or Carob: All Types** (1)<br>Include solid or filled chocolate, chocolate bars, blocks, buttons, M&Ms etc	○	OR	○	○	○	○	○	○	&	○	○	○	○	○	○	○
**Lollies: Sugarfree** (2)<br>Do NOT include "natural" or "no added sugar" lollies	○	OR	○	○	○	○	○	○	&	○	○	○	○	○	○	○
**Lollies That Last a Long Time** (3)<br>Any that take a long time to eat: either because they are sticky and stay in the mouth (e.g. Minties, Red Skins) or because they are hard and have to be sucked, (e.g. lollipops, gobstoppers, toffees etc)	○	OR	○	○	○	○	○	○	&	○	○	○	○	○	○	○
**Lollies: All Other Types** (4)<br>Include anything not already included, such as soft and gummy lollies, liquorice, marshmallow, Skittles, sour worms, party mix etc including "natural" and fruit types	○	OR	○	○	○	○	○	○	&	○	○	○	○	○	○	○

Think about a typical day of eating for your child. On a typical day, how many meals, drinks other than water and snacks does your child eat ?

A meal is generally larger and made of a mixture of different foods. A snack is generally smaller, eaten more quickly and made of only one or two different foods. *Note that some foods may be thought of as a meal OR a snack depending on the time of day and amount eaten. For example, 1 peanut butter sandwich at lunchtime is a meal, half a sandwich in the car at 10am is a snack.*

On a typical day my child has:

☐ meals per day (1)

☐ between meal snacks per day (2)

☐ drinks other than water per day: with food (3)

☐ drinks other than water per day: without food (4)
